# Single nucleotide polymorphisms within HLA region are associated with disease relapse for patients with unrelated cord blood transplantation

**DOI:** 10.7717/peerj.5228

**Published:** 2018-08-02

**Authors:** Ding-Ping Chen, Su-Wei Chang, Tang-Her Jaing, Wei-Ting Wang, Fang-Ping Hus, Ching-Ping Tseng

**Affiliations:** 1 Department of Laboratory Medicine, Chang Gung Memorial Hospital, Taoyuan, Taiwan; 2 Department of Medical Biotechnology and Laboratory Science, College of Medicine, Chang Gung University, Taoyuan, Taiwan; 3 Graduate Institute of Biomedical Sciences, College of Medicine, Chang Gung University, Taoyuan, Taiwan; 4 Division of Allergy, Asthma, and Rheumatology, Department of Pediatrics, Chang Gung Memorial Hospital, Taoyuan, Taiwan; 5 Clinical Informatics and Medical Statistics Research Center, College of Medicine, Chang Gung University, Taoyuan, Taiwan; 6 Division of Hematology and Oncology, Department of Pediatrics, Chang Gung Children’s Hospital, Taoyuan, Taiwan

**Keywords:** Hematopoietic stem cell transplantation, Cord blood transplantation, Human leukocyte antigen, Single nucleotide polymorphisms, Major histocompatibility complex, Linkage disequilibrium

## Abstract

Disease relapse occurs in unrelated cord blood transplantation (CBT) even when the alleles of human leukocyte antigen (HLA) are fully matched between donor and recipient. This is similar to that observed in other types of hematopoietic stem cell transplantation. Fourteen single nucleotide polymorphisms (SNPs) within the HLA region have been reported previously by Petersdorf et al. and Piras et al. as transplantation determinants in unrelated hematopoietic cell transplantation. In this study, the genomic sequences within 500 base pairs upstream and downstream of the fourteen transplantation-related SNPs from 53 patients and their HLA-matched unrelated donors were analyzed for determining whether or not genetic variants, conferred by either recipient or donor SNP genotype or by recipient-donor SNP mismatching, were associated with the risk of relapse. Seven SNPs were associated with the risk of relapse in unrelated CBT. These included the donor genotype with the SNPs of rs2523675 and rs2518028 at the telomeric end of HCP5 gene, rs2071479 in the intron of the HLA-DOB gene, and rs2523958 in the MICD gene; and the recipient genotype with SNPs of rs9276982 in the HLA-DOA gene, and rs435766 and rs380924 in the MICD gene. As measured by pair-wise linkage disequilibrium (LD) with *D*′ as the parameter for normalized standard measurement of LD which compares the observed and expected frequencies of one haplotype comprised by alleles at different loci, rs2523675 had high LD with rs4713466 (*D*′ = 0.86) and rs2523676 (*D*′ = 0.91) in the HCP5 gene. The rs2518028 had no LD with all other SNPs except rs2523675 (*D*′ = 0.76). This study provides the basis for developing a method or algorithm for selecting better unrelated CBT candidate donors.

## Introduction

The human leukocyte antigen (HLA) region that spans 4 × 10^6^ nucleotides of the short arm of chromosome 6 from region 2, band 1, sub-band 1 (6p21.1) to region 2, band 1, sub-band 3 (6p21.3) is the most polymorphic region of the human genome. It is an attractive candidate for discovery of clinically important human genetic variants because high density of immune function-related genes is distributed in this region (Trowsdale, 2011). Hematopoietic stem cell transplantation (HSCT) is effective for treatment of patients with various types of hematologic disorders (Beatty et al., 1985; Saber et al., 2012; Arora et al., 2009; Rocha et al., 2000). Autologous HSCT can be used for enabling very high dose treatment regimens, while allogeneic HSCT, of which cord blood transplantation (CBT) is one variant, can be used for combining high-dose treatment and the allogeneic anti-tumor effect (Ruggeri et al., 1999). Transplantation of patients with HLA-mismatched donors is associated with a high risk of disease relapse, graft-versus-host disease (GVHD) and mortality when compared with HLA-matched donors (Petersdorf et al., 2001; Lee et al., 2007). The outcome of transplantation between related donor-recipient pairs is usually better than that of unrelated pairs (Ho et al., 2011; Uzunel et al., 2006). So choosing related HLA-matched donors in allogeneic transplantation is a priority.

Advances in supportive care have reduced the incidence of complications. However, a significant number of patients still develop life-threatening problems when HLA-matched donors are used in HSCT (Morishima et al., 2002). Other genetic factors beyond HLA alleles affect the outcomes of HSCT. Fourteen single nucleotide polymorphisms (SNPs) within the HLA region have been identified to associate with the risk of mortality, disease-free survival, transplant-related mortality, relapse and acute and chronic GVHD for patients receiving HSCT (Petersdorf et al., 2013; Piras et al., 2014). These include the SNPs with the reference SNP cluster ID (rs), an accession number used by researchers and databases to refer to specific SNP, of rs2244546, rs986522, rs915654, rs429916, rs2242656, rs209130, rs2075800, rs394657, rs2523957, rs3830076, rs2071479, rs11538264, rs10484558, and rs107822. These SNPs either present in the donor DNA, in the recipient DNA, or are mismatched between the donor and the recipient DNA leading to favorable or unfavorable post-HSCT clinical outcome of the recipients (Petersdorf et al., 2013; Piras et al., 2014). Genetic variants spanning HLA loci thereby play a crucial role in the heterogeneous outcome of HSCT (Hansen et al., 2010; Dickinson & Holler, 2008; Conway & Abdi, 2009).

Unrelated CBT is a reliable alternative therapy of HSCT for children and adults with hematologic malignancies (Wagner et al., 1996; Takahashi et al., 2004; Gluckman et al., 1997; Kurtzberg et al., 1996; Laughlin et al., 2004; Rocha et al., 2001). The most important advantage in unrelated CBT is that one or two HLA antigen/allele mismatches between donors and recipients are acceptable for CBT without causing serious adverse effects on the recipients (Kurtzberg et al., 1996; Laughlin et al., 2004; Rocha et al., 2001; Rubinstein et al., 1998; Eapen et al., 2007; Chen et al., 2016). The possibility of finding a donor for patients, where there is difficulty in finding a matched donor, increases with access to CBT.

Disease relapse and/or severe complications may still occur following unrelated CBT despite a high degree of HLA-match by high-resolution sequence-based typing (Chen et al., 2016; Atsuta et al., 2009). In this study, the genomic sequences within 500 base pairs (bp) upstream and downstream of the 14 HSCT-related SNPs (Petersdorf et al., 2013; Piras et al., 2014) for 53 patients and their unrelated HLA-matched donor in CBT were surveyed to determine whether or not genetic variants within the HLA region were associated with the risk of relapse.

## Materials and Methods

### Patients and HLA typing

This study was approved by the Institutional Review Board of Chang Gung Memorial Hospital (CGMH) with the approval ID of 102-4949B. Patients (*n* = 53) with the indicated diseases (Table 1) and undergoing unrelated HLA-matched CBT were recruited at CMGH between 2004 and 2014. The clinical characteristics of these patients are shown in Table 1. All 53 recipients provided written informed consent for participation in this study.

**Table 1:** Clinical characteristic of patients who received unrelated CBT.

Characteristics of patients	Number of patient (%) or median (range)
Number of patients	53
Median age in years (range)	11 (1–23)
Male:Female	34 (64.2%):19 (35.8%)
Diagnosis	
Transfusion-dependent thalassemia	19 (35.8%)
Genetic diseases	10 (18.9%)
Chronic granulomatous disease	1
X-linked chronic granulomatous disease	1
Wiskott–Aldrich syndrome	1
Osteopetrosis	4
Immunodeficiency	3
Acute lymphoid leukemia	6 (11.3%)
Neoplastic diseases	5 (9.4%)
Neuroblastoma	1
Retroperitoneal neuroblastoma	2
Malignant neoplasm	2
Severe aplastic anemia	5 (9.4%)
Fanconi anemia	3 (5.7%)
Acute myeloid leukemia	3 (5.7%)
Chronic myeloid leukemia	2 (3.8%)
Matching at antigen-level for HLA-A and -B and allele-level for HLA-DRB1	
Fully matched	16 (30.2%)
One mismatch	22 (41.5%)
Two mismatches	15 (28.3%)
Three mismatches	0 (0%)
GVHD	27 (50.9%)
Overall survival	44 (83.0%)
Relapse	38 (71.7%)

Prior to transplantation, HLA typing of HLA-A, -C, -B, -DRB1, -DQB1 alleles for donors and patients was performed using the method of LABType SSO Typing Test (Thermo Fisher, Waltham, MA, USA) which was based on the use of sequence-specific oligonucleotide probes. The SeCore kit (Thermo Fisher, Waltham, MA, USA) was then used for high-resolution HLA typing to obtain more detailed allele information. The MicroSSP Allele Specific Typing Tray (Thermo Fisher, Waltham, MA, USA) which was based on the use of sequence-specific primers was used to resolve allele ambiguity of the SeCore typing.

### Engraftment monitoring and relapse evaluation after CBT

Cord blood transplantation engraftment was evaluated by a chimerism test based on short tandem repeats (STR) analysis using the AmpFlSTR Identifiler amplification kit (Thermo Fisher, Waltham, MA, USA). The following tetranucleotide STR loci were included in the STR analysis: D8S1179, D21S11, D7S820, and CSF1PO (all labeled with 6-FAM blue dye); D3S1358, TH01, D13S317, D16S539, and D2S1338 (all labeled with VIC green dye); and D19S3433, vWA, TPOX, and D18S51 (all labeled with NED yellow dye). The PCR cycle conditions and product analysis were performed according to the manufacturer’s instruction. In this study, relapse was defined as recurrence of malignancy based on one or more of the following: bone marrow morphology, minimal residual disease by either flow cytometry, cytogenetics, STR by high-throughput amplicon sequencing or imaging results. Relapse of non-malignant hematological disorders was defined by conversion to nonresponse from partial or complete response.

### Selection of SNPs

The 14 SNPs (rs2244546, rs986522, rs915654, rs429916, rs2242656, rs209130, rs2075800, rs394657, rs2523957, rs3830076, rs2071479, rs107822, rs11538264, and rs10484558) within the HLA region have been reported to associate with the risk of mortality, disease-free survival, transplant-related mortality, relapse and acute and chronic GVHD in patients with HSCT (Petersdorf et al., 2013; Piras et al., 2014). These SNPs were considered in this study as the sourced SNPs. The sourced SNPs were categorized into donor genotype, recipient genotype and donor-recipient genotype based on whether the SNP-associated risks were conferred by either donor or recipient SNP or by donor-recipient SNP mismatching.

The 500 bp genomic regions upstream and downstream of the 14 sourced SNPs were sequenced to search for candidate SNPs that were associated with the risk of relapse in unrelated CBT. A total of 58 SNPs were defined within these regions and were categorized into group 1 (*n* = 19, donor genotype), 2 (*n* = 18, recipient genotype), and 3 (*n* = 21, mismatch between donor-recipient pair) based on the relative position to and the category of the sourced SNPs (Table 2). Whether the SNPs-associated risks were conferred by either donor SNPs (mode of donor genotype analysis), recipient SNP (mode of recipient genotype analysis) or by donor-recipient SNP mismatching (mode of donor-recipient pair analysis) were analyzed.

**Table 2:** The SNPs that were within 500 bps upstream or downstream of the sourced SNPs.

Sourced SNP	Model	SNP under analysis			
rs394657	Donor genotype	rs61365987	rs444472	rs2256594	rs394657
		rs429853	rs111394117	rs568986490	
rs986522	Donor genotype	rs77011831	rs986522	rs115641163	rs986521
		rs2229784			
rs2244546	Donor genotype	rs9281491	rs2244546	rs4713466	rs2523676
		rs2523675	rs2518028	rs141431529	
rs11538264	Recipient genotype	rs543293268	rs17207239	rs1046089	rs532278148
		rs115028652			
rs10484558	Recipient genotype	rs2844463	rs180712068		
rs429916	Recipient genotype	rs9276982	rs71565361	rs79327197	rs151190962
		rs9282369			
rs915654	Recipient genotype	rs2009658	rs736160	rs915654	
rs2075800	Recipient genotype	rs371621895	rs2075800	rs2227956	
rs2242656	Mismatch	rs3130048	rs2844464	rs2242656	
rs3830076	Mismatch	rs3830076			
rs2071479	Mismatch	rs11244	rs2070120	rs41258084	rs17220087
		rs2071479			
rs107822	Mismatch	rs107822	rs213210		
rs2523957	Mismatch	rs435766	rs380924	rs1264813	rs2523960
		rs2523959	rs2523958	rs2523957	rs5009448
rs209130	Mismatch	rs209132	rs209131		

### PCR and sequencing

The recipient and donor DNA from three ml of peripheral blood were extracted by a QIAamp DNA Blood mini Kit (Qiagen, Valencia, CA, USA). A total of 14 different primer pairs (Table 3) were used to amplify the DNA fragments that covered 500 bp upstream and downstream of the 14 sourced SNPs. PCR was performed in a reaction volume of 50 μl containing 1× reaction buffer, 10 nmol of dNTPs, 6 pmol of forward and reverse primers, 300 ng of genomic DNA, and one μl of *Pfu Turbo* Hotstart DNA Polymerase (Agilent, Santa Clara, CA, USA). The cycling condition was 4 min at 94 °C for 1 cycle, 30 s at 94 °C, 30 s at 58 °C, and 45 s at 72 °C for 30 cycles, and 10 min at 72 °C for 1 cycle. Subsequently, five μl of PCR products were fractionated on a 2% agarose gel and visualized by ethidium bromide staining. The remaining PCR product was subject to direct sequencing using the Big Dye Terminator Cycle Sequencing kit (Thermo Fisher, Waltham, MA, USA) and an ABI PRISM Genetic Analyzer (Thermo Fisher, Waltham, MA, USA) according to the manufacturer’s instruction.

**Table 3:** Primer sequences for amplification of candidate SNPs.

Gene	Primer sequence
BAT2 Gene	F: 5′-CACGATGGGGACAGAAAGGT-3′
	R: 5′-TCACTGAAGGGGTCATGCAATG-3′
BAT3 Gene	F: 5′-TCCCACCCATGAGAGGATAG-3′
	R: 5′-TCAGGAGTTCCAATCCAGCCT-3′
BAG6 Gene	F: 5′-ATTCATTCAGGGGCACAAGGGG-3′
	R: 5′-GCGGAGGTTGAAGAGAATAGAAGC-3′
COL11A2 Gene	F: 5′-TGTCCCTCACCTTGGCTCCCTT-3′
	R: 5′-AATTCCTCTCTCCCTAGGGAT-3′
FKBPL Gene	F: 5′-TGATACAACCAGGGCGCTTCAG-3′
	R: 5′-TTGGAGCGGGAGCCTGGCCATTTAAAG-3′
HCP5 Gene	F: 5′-GGGCAACTAAGTCAGGTCTAG-3′
	R: 5′-TCTGCAGGTCTCATGGAGAG-3′
HLA-A Gene	F: 5′-TTCCAAGTGAGGAACTCAGACC-3′
	R: 5′-AAGATGCACTGATCCTCCCT-3′
HLA-DOA Gene	F: 5′-CAACAACGTAAAGCTAACGTCTGTG-3′
	R: 5′-GCACCACTCTTAGTTATGTATAGG-3′
HLA-DOB Gene	F: 5′-TCTTCTGAAGACTGTGGAGACTGC-3′
	R: 5′-TCCCATAGGAGCTCAGTCTGAAT-3′
HSPA1L Gene	F: 5′-TCCCCTTCAAGGTACATTCACAGCC-3′
	R: 5′-TGATCCAGGTGTATGAGGGCGAGAG-3′
LTA Gene	F: 5′-AGCATAAAAGGCAAAGGGGCAG-3′
	R: 5′-TTAGGTATGAGGTGGACACCTC-3′
NOTCH4 Gene	F: 5′-GATTGTCTGTTGGGTGACCTGAG-3′
	R: 5′-TGAGGCTGATCACAATGAGTGCCTCTC-3′
RING1 Gene	F: 5′-TAATCGACTCTGGCGCCCACAT-3′
	R: 5′-AACAACCTTAGCCTCGGTTCCCTT-3′
TRIM27 Gene	F: 5′-AGTCGGGATTACAGAAATGCACC-3′
	R: 5′-GCAGGACATTTGAAGGTAACC-3′

### Statistical analysis

The Hardy-Weinberg equilibrium (HWE) test was performed to examine the quality of experiments for the tested SNPs. SNPs that violated the HWE were eliminated from analysis. The allele and genotype frequencies were calculated and compared between relapse and non-relapse groups to evaluate the association between disease relapse and candidate SNPs. The PLINK software v1.07 was used to carry out logistic regression under four types of models assuming different allele effects: allelic, additive, dominant and recessive (Purcell et al., 2007). A genotypic test was carried out to investigate the association of specific SNP genotypes with disease relapse. The association between disease relapse and mismatch status of SNP genotypes in donor-recipient pairs was examined using the chi-square and Fisher’s exact tests. Logistic regression was also performed to investigate the SNP association with and without adjustment of age and sex. Since the age and sex of the donor-recipient pairs had no effect, we only showed the results of the logistic regression analysis assuming different allele effects without any adjustment in Tables S4 and S5. The measurement of pair-wise linkage disequilibrium (LD) for the SNPs in groups 1, 2, and 3 which refers to the non-random association of alleles at two or more loci in a general population was determined by using HaploView 4.2 (https://www.broadinstitute.org/haploview/haploview) (Barrett et al., 2005).

## Results

A total of 58 SNPs were evaluated for determination whether or not any of them were associated with the risk of relapse among 53 donor-recipient pairs of unrelated CBT. Group 1 and 3 SNPs were subject to donor genotype analysis, groups 2 and 3 SNPs were subject to recipient genotype analysis and group 3 SNPs were subject to donor-recipient pair analysis (Tables S1–S3; Fig. S1).

Of the 19 SNPs in group 1, seven SNPs were located in the intron of the NOTCH4, 5 SNPs were located in the intron of the COL11A2, and seven SNPs were at the telomeric end of the HLA class I histocompatibility antigen protein P5 (HCP5) gene, respectively (Table S4). Donor genotype analysis of all 19 SNPs revealed that two of the SNPs, rs2523675, and rs2518028, located at the telomeric end of HCP5 gene were associated with the risk of relapse (genotypic test: *P* = 0.0268 and 0.0233, respectively) (Table 4 and Table S4). The number of T alleles of rs2523675 was showed to be positively associated with the risk of relapse. The donors who carried the polymorphism of T at rs2523675 resulted in 2.75 times greater risk of relapse for the recipients than the donors who carried the polymorphism of C in the same SNP position (allelic model: *P* = 0.0328, 95% CI of OR = 1.09–6.93). On the other hand, the donor who carried the G/G allele in rs2518028 resulted in 4.52 times greater risk of relapse for the recipients than the donors who carried the A/G or A/A alleles (recessive model: *P* = 0.0272, 95% CI of OR = 1.19–17.13) (Table S4).

**Table 4:** The donors and recipient types SNPs that are associated with the risk of relapse for patients with unrelated CBT.

Type	SNP	Genome position^1^ (bp)	Gene/location	Source^2^	Number of patients (%)			*P*
Donor								
	rs2523675	31468255	2.4 kb telomeric of HCP5	rs2244546	CC	CT	TT	0.0268
	relapse				13 (35.1)	11 (29.7)	13 (35.1)	
	non-relapse				7 (46.7)	8 (53.3)		
	rs2518028	31468270	2.5 kb telomeric of HCP5	rs2244546	AA	AG	GG	0.0233
	relapse				1 (2.7)	5 (13.5)	31 (83.8)	
	non-relapse					7 (46.7)	8 (53.3)	
	rs2071479	32813335	HLA-DOB, intron	rs2071479	CC	CT	–	0.0077
	relapse				35 (100)	0 (0)		
	non-relapse				11 (73.3)	4 (26.7)		
	rs2523958	29972425	MICD	rs2523957	CC	CT	TT	0.0445
	relapse				26 (72.2)	6 (16.7)	4 (11.1)	
	non-relapse				8 (53.3)	7 (46.7)		
Recipient								
	rs9276982	33010438	HLA-DOA, promoter	rs429916	AA	AG	GG	0.0376
	relapse				1 (2.6)	7 (18.4)	30 (79.0)	
	non-relapse					8 (53.3)	7 (46.7)	
	rs435766	29972075	MICD	rs2523957	CC	CT	TT	0.0130
	relapse				14 (37.8)	11 (29.7)	12 (32.4)	
	non-relapse				5 (33.3)	10 (66.7)	0 (0)	
	rs380924	29972108	MICD	rs2523957	CC	CT	TT	0.0320
	relapse				12 (32.4)	11 (29.7)	14 (37.8)	
	non-relapse				1 (6.7)	10 (66.7)	4 (26.7)	

**Notes:**

1 Assembly version: GRCh37.p13.

2 The SNPs were selected and studied based on the transplant determinants identified by Petersdorf et al. (2013).

Of the 18 SNPs in group 2, five SNPs were located in the intron or exon of HLA-B associated transcript (BAT2), two SNPs were located in the intron of BAT3, five SNPs were located at the centromeric end of HLA-DOA, three SNPs were located at the telomeric end of lymphotoxin-alpha, and three SNPs were located in the exon of heat shock protein family A member 1 like genes, respectively (Table S5). Recipient genotype analysis of all 18 SNPs revealed that the SNP of rs9276982 located at the centromeric end of HLA-DOA gene was associated with the risk of relapse (genotypic test: *P* = 0.0376; Table 4). Disease relapse for recipients who carried two G alleles in rs9276982 were 4.29 times greater than those who carried only one G allele or A alleles (recessive model: *P* = 0.0258, 95% CI of OR = 1.2–15.31) (Table S5).

Of the 21 SNPs in group 3, three SNPs were located in the intron of BAG6, one SNP was at the telomeric end of FKBPL, three SNPs were in the intron or exon of HLA-DOB, two SNPs were at the telomeric end of RING1, eight SNPs were in the MICD gene, and two SNPs at the telomeric end of TRIM27 genes, respectively (Table S6). Analysis of mismatch between donor-recipient pair genotype SNPs revealed that none were associated with the risk of relapse (Table S6), while analysis of donor genotype SNPs (Table S7) revealed that rs2071479 located in the intron of HLA-DOB gene and rs2523958 located in the MICD gene were associated with disease relapse (rs2071479: genotypic test *P* = 0.0077; rs2523958: genotypic test *P* = 0.0445; Table 4). All donors and 72.2% of the donors in the relapse group had the CC genotype of rs2071479 and rs2523978, respectively. Recipient genotype analysis of these SNPs (Table S7) revealed that rs435766 and rs380924 located in the MICD gene (genotypic test *P* = 0.0130 and 0.0320, respectively) were associated with disease relapse (Table 4). Two-thirds of recipients (66.7%) in the non-relapse group had the CT genotype of rs435766 and rs380924, while no genotype was associated with recipients in the relapse group.

In the group 1 SNPs, rs2523675 had a high LD with rs4713466 (*D*′ = 0.86) and rs2523676 (*D*′ = 0.91) located in the HCP5 gene (Fig. S2), of which *D*′ is the normalized standard measurement of LD by comparing the observed and expected frequencies of one haplotype comprised by alleles at different loci. The SNP of rs2518028 had no LD with all other SNPs except rs2523675 (*D*′ = 0.76). In the group 2 SNPs, only rs180712068 in the BAT3 gene had a high LD with rs115028652 (*D*′ = 1) and rs1046089 (*D*′ = 0.92) in the BAT2 gene (Fig. S3). In the group 3 SNPs, rs1264813, and rs2523960 in HLA-A showed a complete LD with each other, and so did the three SNPs, rs2070120, rs41258084, and rs17220087 in HLA-DOB (Fig. S4).

## Discussion

**Figure 1:**
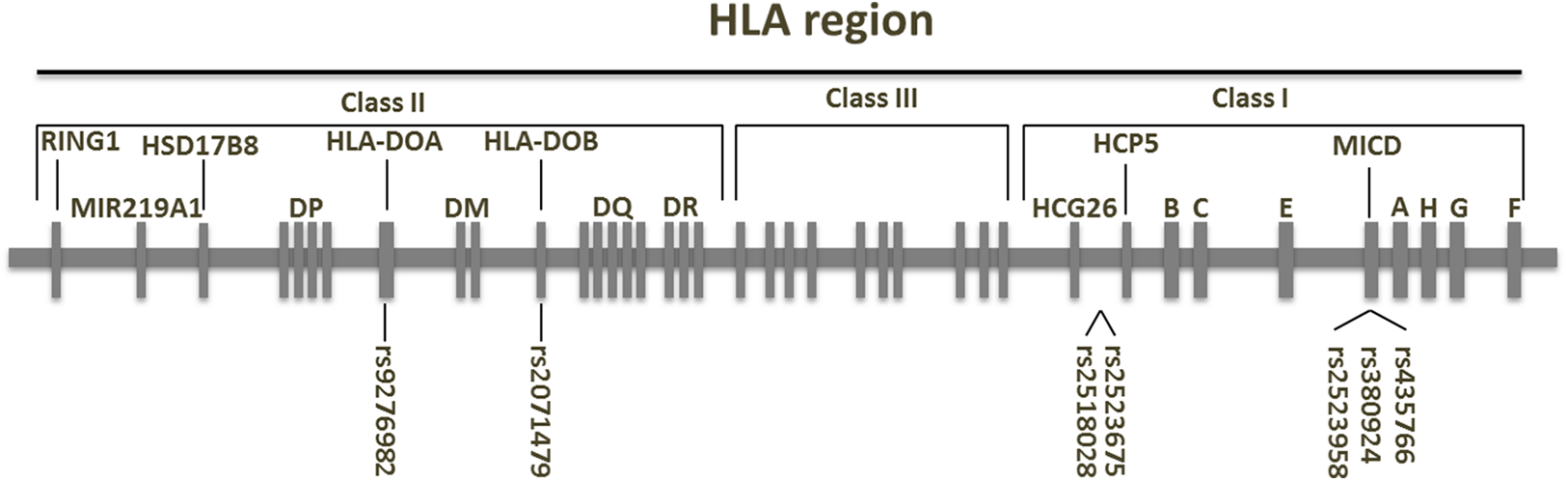
Relative position of the SNPs associated with the risk of relapse after unrelated CBT. Seven SNPs that are associated with the risk of relapse after unrelated CBT are shown on a map of MHC on chromosome 6p21.3. SNPs are identified by their rs numbers.

A panel of SNPs within the HLA region was associated with the risk of relapse following analysis of 53 donor-recipient pairs of CBT. These include the donor type SNPs: rs2523675 and rs2518028 at the telomeric end of HCP5 gene; rs2071479 in the intron of the HLA-DOB gene; and rs2523958 in the MICD gene; and the recipient type SNPs: rs9276982 in the HLA-DOA gene, and rs435766 and rs380924 in the MICD gene (Fig. 1). This represents the first report relating SNPs in the HLA region with the risk of relapse of CBT.

**Figure 2:**
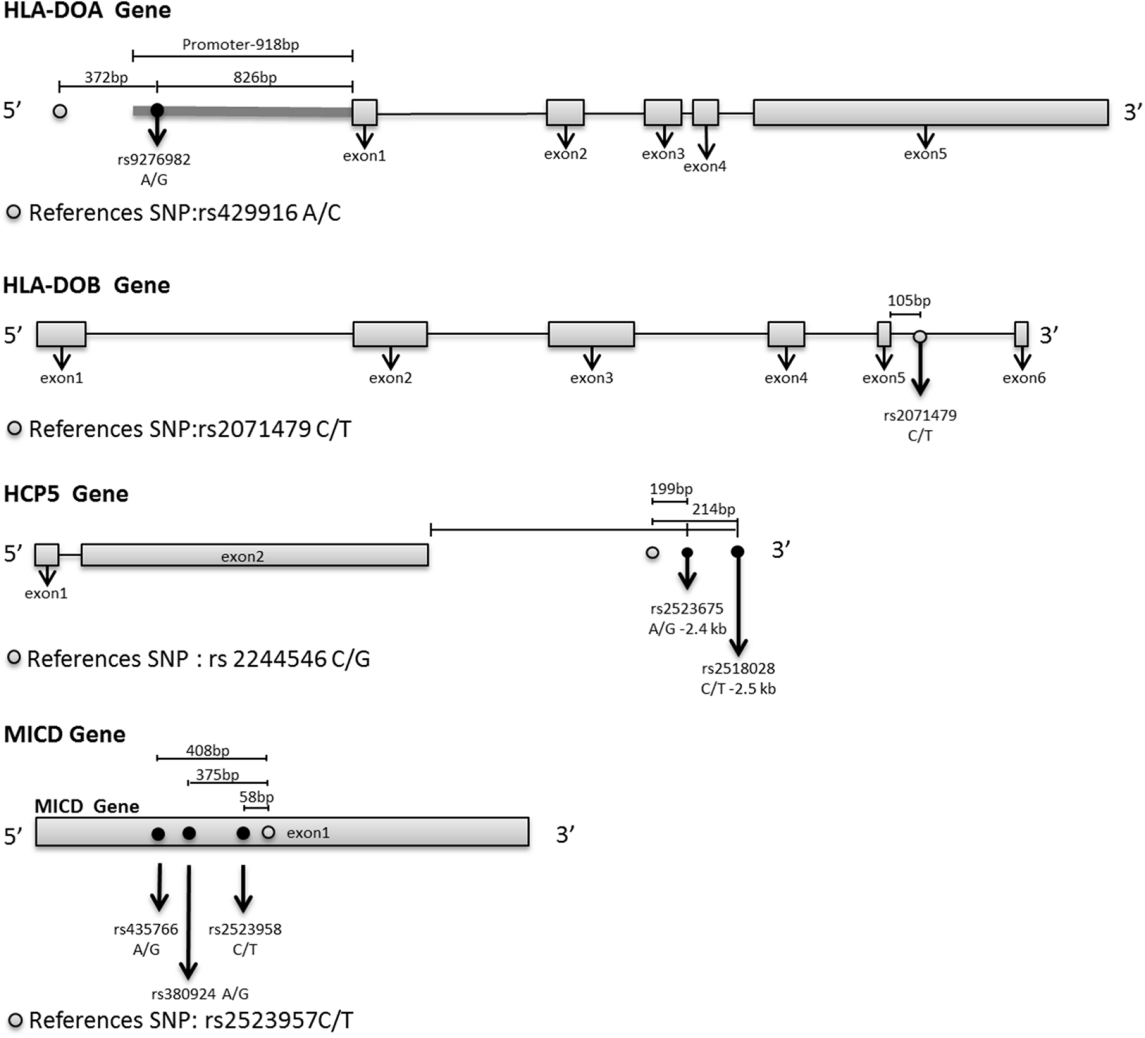
The relative positions of the relapse-associated SNPs to the indicated genes. The structure for the indicated genes nearby the relapse-associated SNPs is shown. Reference SNP is the sourced SNP as reported previously.

There are about five to ten cases of CBT per year at CGMH (Jaing et al., 2012). The 53 donor-recipient pairs of CBT represent a collection of specimen over a period of nine years. The SNPs associated with the risk of relapse after HLA-matched unrelated CBT were mainly on or flanking the genomic sequences of the HCP5, HLA-DOA, HLA-DOB, and MICD genes. rs2523675 and rs2518028 were located at 2,446 and 2,461 bp from 3′ of the HCP5 exon 2 (Fig. 2). HCP5 is localized within the HLA class I region, but is not structurally related to the HLA class I genes (Vernet et al., 1993). Multiple copies of the short coding region are present in the genomic region of HCP5. P5-1 is one of the HCP5 family members encoding a peptide of 52 amino acids with a domain identical in sequence to the signal peptide of HLA molecules (Kulski & Dawkins, 1999). The transcript of P5-1 is composed of the 5′ sequence of an HLA class I gene including the promoter region, the first exon, and the half of the first intron fused to an unrelated intron, followed by a large exon. P5-1 is specifically expressed in lymphoid cells and tissues, suggesting an immunological function for the protein product of HCP5 gene (Avoustin et al., 1994).

The recipient type SNP, rs9276982 and the donor type SNP, rs2071479 were located in the promoter region of the HLA-DOA and the intron of the HLA-DOB gene, respectively. HLA-DOA belongs to the HLA class II alpha chain paralogues and forms a heterodimer with HLA-DOB (Naruse et al., 2002). The heterodimer, HLA-DO, functions as an HLA class II molecular chaperone in modulating antigen presentation and regulating HLA-DM-mediated peptide loading on HLA class II molecules. HLA-DO dysfunction leads to less-restrictive antigen presentation (van Ham et al., 1997). The presentation of DM-sensitive antigens is decreased by HLA-DM and restored by co-expression of HLA-DO (Bellemare-Pelletier et al., 2005). In comparison with other classical HLA class II molecules, HLA-DOA exhibits little sequence variation, particularly at the protein level (Naruse et al., 2002). The association of rs9276982 in the promoter region of HLA-DOA with the risk of relapse after unrelated CBT suggests that rs9276982 may affect the expression of HLA-DOA and subsequently the formation of HLA-DO. The SNP, rs2071479 that was present in the intron of HLA-DOB may cause inaccurate mRNA splicing and generate protein variants with impaired function (Yu et al., 2009). HLA-DM-mediated HLA class II molecules loaded with antigenic peptides followed by antigen presentation to CD4^+^ T cells is important in the generation of adaptive immune response (Souwer et al., 2009). This process is negatively modulated by the interaction of HLA-DM with HLA-DO. The HLA-DOB variant SNP rs2071479 may lose its inhibitory activity on HLA-DM, leading to an increase in the risk of relapse after unrelated HLA-matched CBT.

The donor type SNP, rs2523958 and the recipient type SNPs, rs435766 and rs380924 were all located in the MICD gene. In humans, MICD is a pseudogene due to debilitating mutations or deletions (Bahram & Spies, 1996) and belongs to the MICs gene family, which includes MICA, MICB, MICD, and MICE. Although MICD is a pseudogene, SNPs in MICD are associated with various diseases. The SNP rs5009448 located in the MICD gene loci is among the frequent loss of heterozygosity loci at 6p in nasopharyngeal carcinoma in southern China specifically linked to Epstein Barr virus etiopathogenesis (Li et al., 2013). The SNPs rs2523946 and rs3823355 in the MICD gene loci are associated with multiple sclerosis susceptibility and are in LD with the SNP of rs4959039 (Cree et al., 2010). It is not clear why SNPs in the MICD pseudogene are associated with the risk of relapse after unrelated CBT or with the susceptibility of other diseases. Whether or not the SNPs in the MICD pseudogene have a genetic linkage with other nearby SNPs located in functional gene loci waits to be elucidated. The likelihood that MICD has an unknown functional effect on CBT cannot be ruled out.

The design of the current study was based on the findings by Petersdorf et al. (2013) and Piras et al. (2014) who analyzed the association of SNPs with the outcome of HSCT. However, different SNPs were found to pose as risk factors for relapse after CBT and other subtypes of HSCT. Ethnic difference is a possible explanation for these observations. Similar to this notion, the SNP rs4349859 strongly tagged HLA-B*27 and is a hallmark for all major European ankylosing spondylitis-related subtypes (The Australo-Anglo-American Spondyloarthritis Consortium (TASC) et al., 2011). A genome-wide association study has revealed that rs13202464, instead of rs4349859, within the MHC region represents the main risk effect of HLA-B*27 variants in Han Chinese (Cortes et al., 2013). In addition to the SNPs flanking the HLA loci, many SNPs beyond chromosome 6, where the HLA loci are located, are related to relapse after HSCT (Dickinson & Charron, 2005; Qin et al., 2016; Wun et al., 2017; Berro et al., 2017). The SNPs within the tumor necrosis factor II receptor superfamily member 1B gene and the interleukin 10 gene in human chromosome 1 are associated with improved survival after HSCT (Dickinson et al., 2010).

The current findings may have an impact on the future practice of unrelated CBT. A 50% match of HLA between donor and recipient is acceptable for CBT (Chen et al., 2016). This increases the availability and the number of appropriate donors for CBT.

## Conclusion

A panel of seven SNPs in the HLA regions was associated with the risk of relapse in CBT. This study may provide a basis for the development of a screening panel of SNPs for seeking donors and might lead to a better strategy for searching and selecting alternative donors for transplantation. Because the genes adjacent to these SNPs are related to immunological functions or the susceptibility to the immunological disorders, future studies clarifying the effects of these SNPs on the biological functions of the adjacent genes may contribute to elucidating the mechanism of transplantation failure.

## Supplemental Information

10.7717/peerj.5228/supp-1 Supplemental Information 1 Fig. S1. SNP sequence. The sequence of rs2071479 within HLA-DOB gene, rs2523675 & rs2518028 within HCP5 gene, rs9276982 within HLA-DOA gene, rs435766, rs380924 & rs2523958 within MICD gene.

10.7717/peerj.5228/supp-2 Supplemental Information 2 Fig. S2. LD plot for the group 1 SNPs. The D’ measures between the pair of 19 SNPs on NOTCH4, COL11A2 and HCP5 were calculated using the software HaploView 4.2.

10.7717/peerj.5228/supp-3 Supplemental Information 3 Fig. S3. LD plot for the group 2 SNPs. The D’ measures between the pairs of 18 SNPs on BAT2, BAT3, HLA-DOA, HCP5 SNPs and HSPA1L were calculated using the software HaploView 4.2.

10.7717/peerj.5228/supp-4 Supplemental Information 4 Fig. S4. LD plot for the group 3 SNPs. The D’ measures between the pair of 21 SNPs on BAG6, FKBPL, HLA-DOB, RING1, HLA-A, and TRIM27 were calculated using the software HaploView 4.2.

10.7717/peerj.5228/supp-5 Supplemental Information 5 Table S1. SNP raw data of group 1.

10.7717/peerj.5228/supp-6 Supplemental Information 6 Table S2. SNP raw data of group 2. The SNP raw data of recipient within HLA-DOA, BAT2, BAT3, LTA & HSPA1L gene.

10.7717/peerj.5228/supp-7 Supplemental Information 7 Table S3. SNP raw data of group 3. The SNP raw data of donor & recipient within MICD, HLA-DOB, BAG6, FKBPL, RING1 & TRIM27 gene.

10.7717/peerj.5228/supp-8 Supplemental Information 8 Table S4. Analysis of group 1 SNPs.

10.7717/peerj.5228/supp-9 Supplemental Information 9 Table S5. Analysis of group 2 SNPs. The association of group 2 SNPs with the risk of relapse for patients with unrelated CBT exact test.

10.7717/peerj.5228/supp-10 Supplemental Information 10 Table S6. Analysis of group 3 SNPs. The association of group 3 SNPs with the risk of relapse for patients with unrelated CBT as analyzed by Chi-square test and Fisher’s exact test.

10.7717/peerj.5228/supp-11 Supplemental Information 11 Table S7. Analysis of group 3 SNPs. The association of group 3 SNPs with the risk of relapse for patients with unrelated CBT as analyzed by genotypic test.

## Additional Information and Declarations

### Data Availability

The following information was supplied regarding data availability: The raw data are included in the Supplemental Files.
