# Supplementary figures and images for: Single nucleotide polymorphisms within HLA region are associated with disease relapse for patients with unrelated cord blood transplantation
Source: PeerJ. 2018 Aug 2;6:e5228. doi: 10.7717/peerj.5228 (PMC6076982; doi:10.7717/peerj.5228)

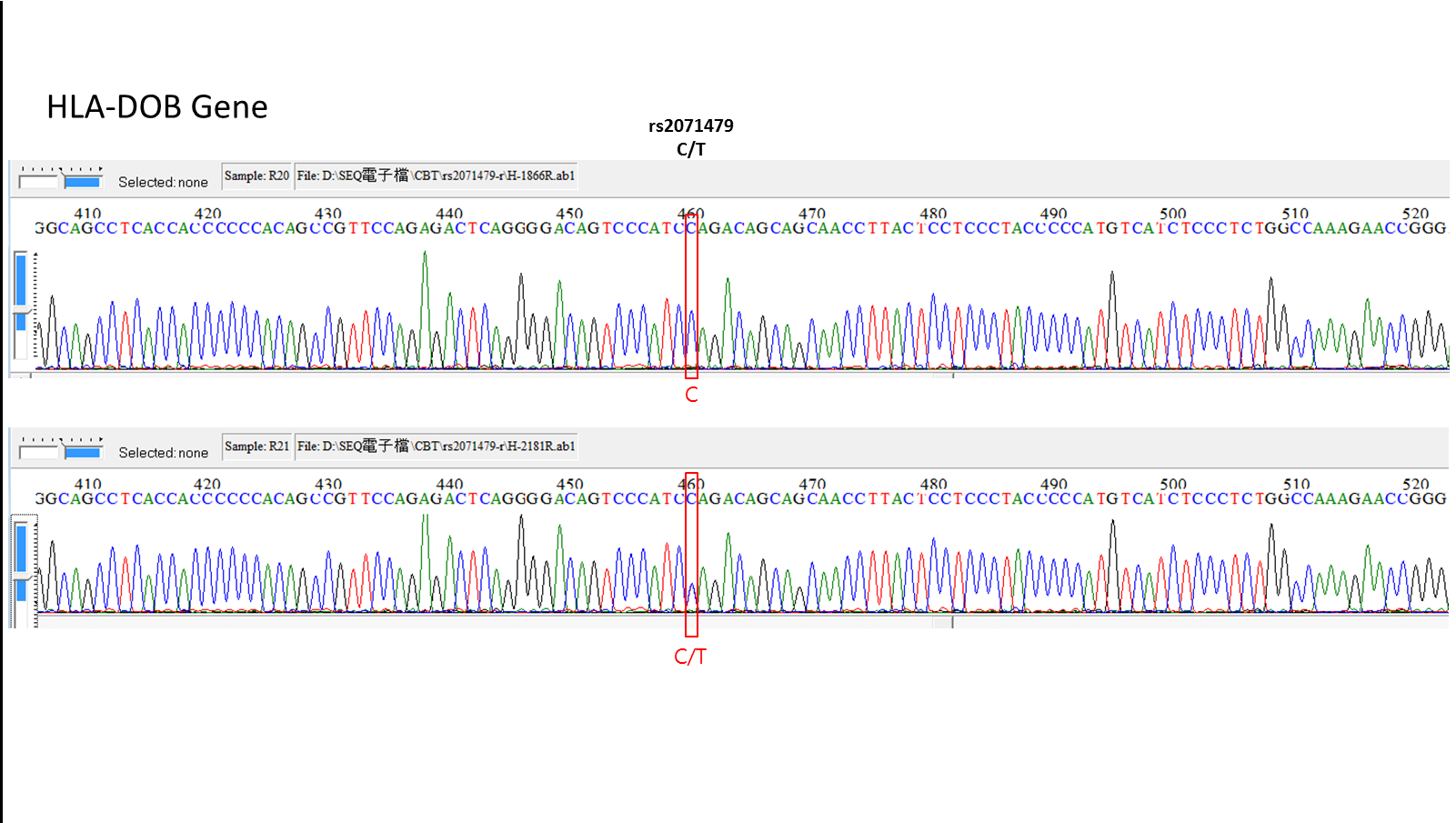

Supplement: Supplemental Information 1 — The sequence of rs2071479 within HLA-DOB gene, rs2523675 & rs2518028 within HCP5 gene, rs9276982 within HLA-DOA gene, rs435766, rs380924 & rs2523958 within MICD gene. [file peerj-06-5228-s001.png]

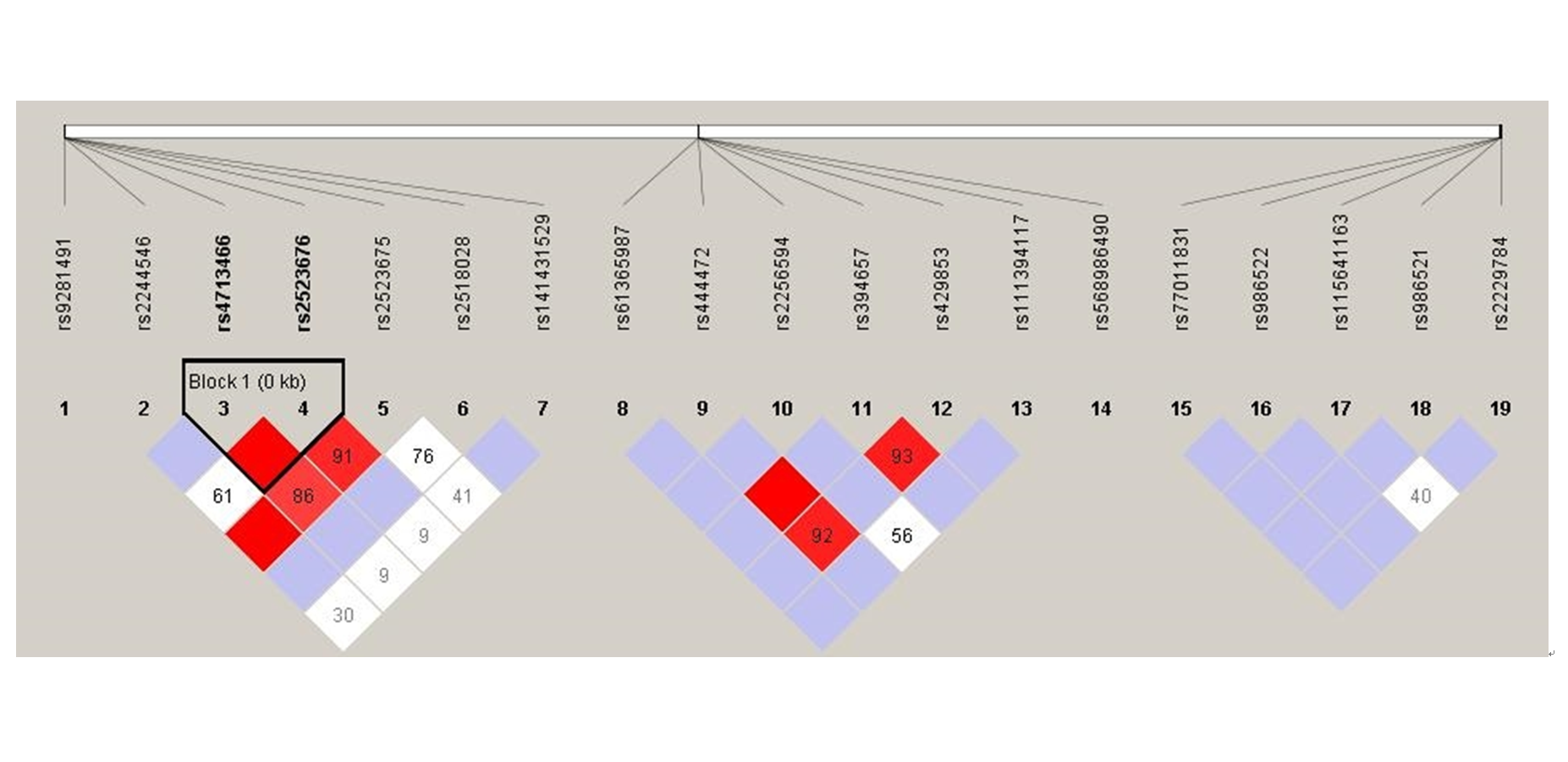

Supplement: Supplemental Information 2 — The D’ measures between the pair of 19 SNPs on NOTCH4, COL11A2 and HCP5 were calculated using the software HaploView 4.2. [file peerj-06-5228-s002.png]

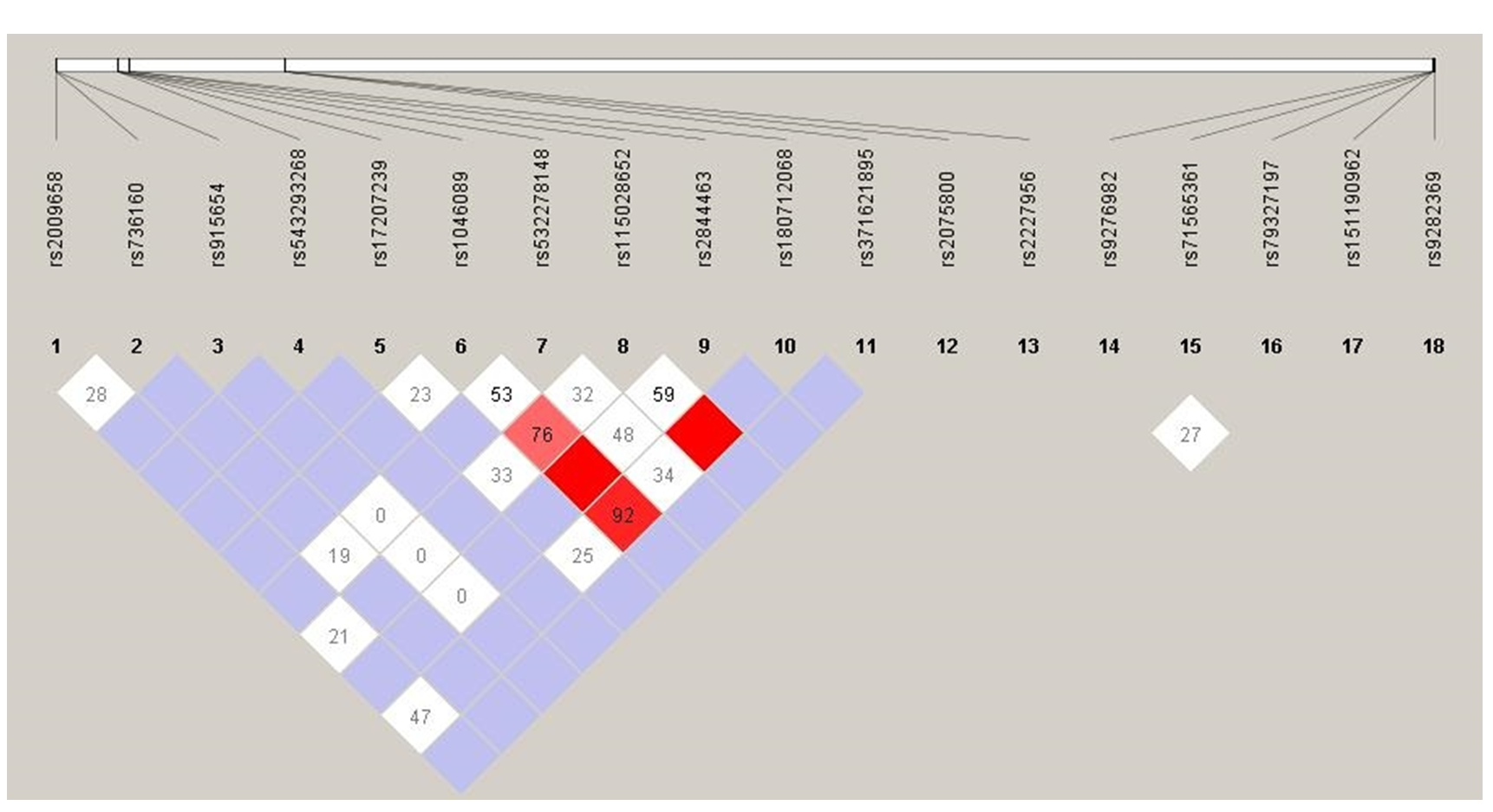

Supplement: Supplemental Information 3 — The D’ measures between the pairs of 18 SNPs on BAT2, BAT3, HLA-DOA, HCP5 SNPs and HSPA1L were calculated using the software HaploView 4.2. [file peerj-06-5228-s003.png]

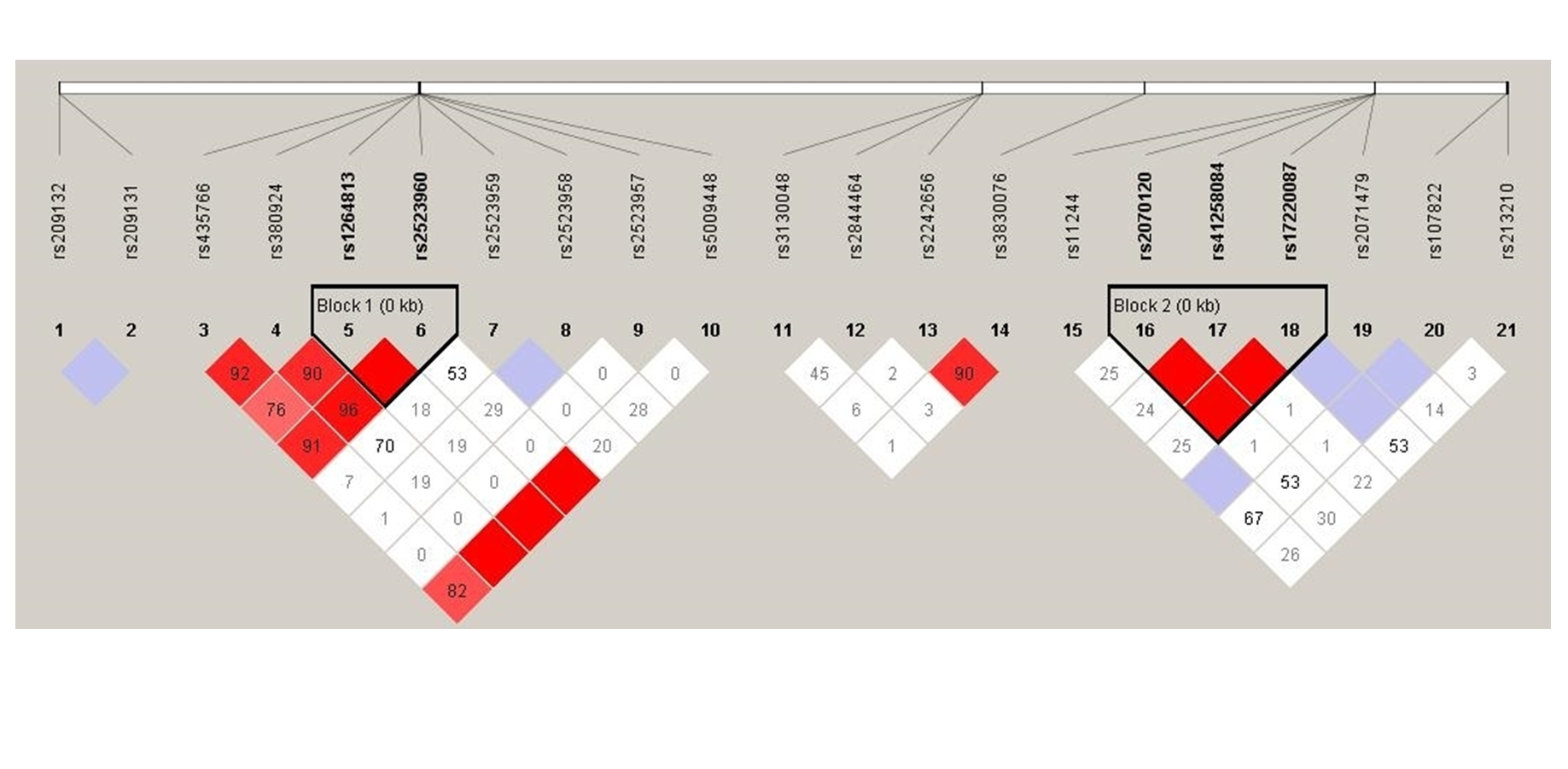

Supplement: Supplemental Information 4 — The D’ measures between the pair of 21 SNPs on BAG6, FKBPL, HLA-DOB, RING1, HLA-A, and TRIM27 were calculated using the software HaploView 4.2. [file peerj-06-5228-s004.png]
