# Supplementary material for: Single nucleotide polymorphisms within HLA region are associated with disease relapse for patients with unrelated cord blood transplantation
Source: PeerJ. 2018 Aug 2;6:e5228. doi: 10.7717/peerj.5228 (PMC6076982; doi:10.7717/peerj.5228)
Supplement: Supplemental Information 8 [file peerj-06-5228-s008.docx]

Supplementary Table S4. The association of group 1 SNPs with the risk of relapse for patients with unrelated CBT.

| **SNP** | **Physical Position^1^ (bp)** | **Gene/location** | **Source^2^** | **Allele Frequency**  **Number of allele (%)** | **Genotype Frequency**  **Number of patients (%)** | | | **P** | **Model** | **P** | **OR** |
| --- | --- | --- | --- | --- | --- | --- | --- | --- | --- | --- | --- |
|  |  |  |  |  |  | | |  |  |  |  |
| rs61365987 | 32218864 | NOTCH4, | rs394657 | C | CC | CT | TT | 0.1263 | Allelic | 0.1277 | 4.20 (0.67-26.37) |
| Relapse |  | intron |  | 70 (97.2) | 34 (94.4) | 2 (5.6) | . |  | Additive | 0.1162 | 4.64 (0.69-31.13) |
| Non-relapse |  |  |  | 25 (89.3) | 11 (78.6) | 3 (21.4) | . |  | Dominant | N/A | N/A |
|  |  |  |  |  |  |  |  |  | Recessive | 0.1162 | 4.64 (0.69-31.13) |
| rRs444472 | 32218949 | NOTCH4, | rs394657 | A | AA | AG | GG | 0.8869 | Allelic | 0.5483 | 1.45 (0.44-4.82) |
| Relapse |  | intron |  | 14 (19.4) | 2 (5.6) | 10 (27.8) | 24 (66.7) |  | Additive | 0.5589 | 1.42 (0.44-4.64) |
| Non-relapse |  |  |  | 4 (14.3) | . | 4 (28.6) | 10 (71.4) |  | Dominant | 0.7461 | 1.25 (0.33-4.79) |
|  |  |  |  |  |  |  |  |  | Recessive | 0.9745 | N/A |
| rs2256594 | 32219095 | NOTCH4, | rs394657 | T | CC | CT | TT | 1.0000 | Allelic | 0.7190 | 1.18 (0.47-2.95) |
| Relapse |  | intron |  | 49 (68.1) | 4 (11.1) | 15 (41.7) | 17 (47.2) |  | Additive | 0.7257 | 1.17 (0.48-2.87) |
| Non-relapse |  |  |  | 18 (64.3) | 2 (14.3) | 6 (42.9) | 6 (42.9) |  | Dominant | 0.7570 | 1.33 (0.22-8.17) |
|  |  |  |  |  |  |  |  |  | Recessive | 0.7811 | 1.19 (0.35-4.11) |
| rs394657 | 32219246 | NOTCH4, | rs394657 | G | AA | AG | GG | 0.6564 | Allelic | 0.3769 | 1.71 (0.52-5.63) |
| Relapse |  | intron |  | 16 (22.2) | 22 (61.1) | 12 (33.3) | 2 (5.6) |  | Additive | 0.3781 | 1.72 (0.52-5.72) |
| Non-relapse |  |  |  | 4 (14.3) | 10 (71.4) | 4 (28.6) | . |  | Dominant | 0.4969 | 1.59 (0.42-6.03) |
|  |  |  |  |  |  |  |  |  | Recessive | 0.9745 | N/A |
| rs429853 | 32219425 | NOTCH4, | rs394657 | C | CC | CT | TT | 0.6738 | Allelic | 0.3769 | 1.71 (0.52-5.63) |
| Relapse |  | intron |  | 16 (22.2) | 3 (8.3) | 10 (27.8) | 23 (63.9) |  | Additive | 0.4062 | 1.63 (0.52-5.09) |
| Non-relapse |  |  |  | 4 (14.3) | . | 4 (28.6) | 10 (71.4) |  | Dominant | 0.6142 | 1.41 (0.37-5.38) |
|  |  |  |  |  |  |  |  |  | Recessive | 0.9687 | N/A |
| rs111394117 | 32219436 | NOTCH4, | rs394657 | A | AA | AG | GG | 0.703 | Allelic | 0.9762 | N/A |
| Relapse |  | intron |  | 4 (5.6) | 1 (2.8) | 2 (5.6) | 33 (91.7) |  | Additive | 0.9628 | N/A |
| Non-relapse |  |  |  | . | . | . | 14 (100.0) |  | Dominant | 0.9687 | N/A |
|  |  |  |  |  |  |  |  |  | Recessive | 0.9820 | N/A |
| rs568986490 | 32219500 | NOTCH4, | rs394657 | T | CC | CT | TT | N/A | Allelic | N/A | N/A |
| Relapse |  | intron |  | . | 36 (100.0) | . | . |  | Additive | N/A | N/A |
| Non-relapse |  |  |  | . | 14 (100.0) | . | . |  | Dominant | N/A | N/A |
|  |  |  |  |  |  |  |  |  | Recessive | N/A | N/A |
| rs77011831 | 33167964 | COL11A2, | rs986522 | C | CC | CT | TT | 0.0782 | Allelic | 0.9823 | N/A |
| Relapse |  | intron |  | 74 (100.0) | 37 (100.0) | . | . |  | Additive | 0.9727 | N/A |
| Non-relapse |  |  |  | 28 (93.3) | 13 (86.7) | 2 (13.3) | . |  | Dominant | N/A | N/A |
|  |  |  |  |  |  |  |  |  | Recessive | 0.9727 | N/A |
| rs986522 | 33168185 | COL11A2, | rs986522 | G | AA | AG | GG | 1.0000 | Allelic | 0.9684 | 1.02 (0.38-2.77) |
| Relapse |  | intron |  | 57 (77.0) | 3 (8.1) | 11 (29.7) | 23 (62.2) |  | Additive | 0.9703 | 1.02 (0.4-2.6) |
| Non-relapse |  |  |  | 23 (76.7) | 1 (6.7) | 5 (33.3) | 9 (60.0) |  | Dominant | 0.8600 | 0.81 (0.08-8.36) |
|  |  |  |  |  |  |  |  |  | Recessive | 0.8846 | 1.10 (0.32-3.72) |
| rs115641163 | 33168299 | COL11A2, | rs986522 | G | AA | AG | GG | 1.0000 | Allelic | 0.5191 | 2.52 (0.15-41) |
| Relapse |  | intron |  | 73 (98.7) | . | 1 (2.7) | 36 (97.3) |  | Additive | 0.5146 | 2.57 (0.15-43.35) |
| Non-relapse |  |  |  | 29 (96.7) | . | 1 (6.7) | 14 (93.3) |  | Dominant | N/A | N/A |
|  |  |  |  |  |  |  |  |  | Recessive | 0.5146 | 2.57 (0.15-43.35) |
| rs986521 | 33168368 | COL11A2, | rs986522 | A | AA | AG | GG | 0.2928 | Allelic | 0.9875 | N/A |
| Relapse |  | intron |  | 74 (100.0) | 37 (100.0) | . | . |  | Additive | 0.9808 | N/A |
| Non-relapse |  |  |  | 29 (96.7) | 14 (93.3) | 1 (6.7) | . |  | Dominant | N/A | N/A |
|  |  |  |  |  |  |  |  |  | Recessive | 0.9808 | N/A |
| rs2229784 | 33168533 | COL11A2, | rs986522 | A | AA | AC | CC | 0.7154 | Allelic | 0.6481 | 1.46 (0.29-7.42) |
| Relapse |  | intron |  | 7 (9.5) | . | 7 (18.9) | 30 (81.1) |  | Additive | 0.6314 | 1.52 (0.28-8.24) |
| Non-relapse |  |  |  | 2 (6.7) | . | 2 (13.3) | 13 (86.7) |  | Dominant | 0.6314 | 1.52 (0.28-8.24) |
|  |  |  |  |  |  |  |  |  | Recessive | N/A | N/A |
| rs9281491 | 31468038 | 2.2 kb telomeric | rs2244546 | A | AA | A- | -- | 0.1393 | Allelic | 0.0605 | 2.80 (0.96-8.16) |
| Relapse | 31468039 | of HCP5 |  | 28 (37.8) | 9 (24.3) | 10 (27.0) | 18 (48.7) |  | Additive | 0.1292 | 2.01 (0.82-4.96) |
| Non-relapse |  |  |  | 5 (17.9) | 2 (14.3) | 1 (7.1) | 11 (78.6) |  | Dominant | 0.0636 | 3.87 (0.93-16.06) |
|  |  |  |  |  |  |  |  |  | Recessive | 0.4421 | 1.93 (0.36-10.21) |
| rs2244546 | 31468056 | 2.2 kb telomeric | rs2244546 | C | CC | CG | GG | 0.7631 | Allelic | 0.8493 | 1.15 (0.28-4.76) |
| Relapse |  | of HCP5 |  | 67 (90.5) | 31 (83.8) | 5 (13.5) | 1 (2.7) |  | Additive | 0.8571 | 1.13 (0.29-4.35) |
| Non-relapse |  |  |  | 25 (89.3) | 11 (78.6) | 3 (21.4) | . |  | Dominant | 0.9822 | N/A |
|  |  |  |  |  |  |  |  |  | Recessive | 0.6639 | 1.41 (0.3-6.57) |
| rs4713466 | 31468092 | 2.3 kb telomeric | rs2244546 | C | CC | CT | TT | 0.3278 | Allelic | 0.5582 | 1.34 (0.5-3.57) |
| Relapse |  | of HCP5 |  | 57 (77.0) | 22 (59.5) | 13 (35.1) | 2 (5.4) |  | Additive | 0.5351 | 1.40 (0.49-3.99) |
| Non-relapse |  |  |  | 20 (71.4) | 6 (42.9) | 8 (57.1) | . |  | Dominant | 0.9747 | N/A |
|  |  |  |  |  |  |  |  |  | Recessive | 0.2913 | 1.96 (0.57-6.75) |
| rs2523676 | 31468214 | 2.4 kb telomeric | rs2244546 | G | AA | AG | GG | 0.5933 | Allelic | 0.6556 | 1.23 (0.5-3.07) |
| Relapse |  | of HCP5 |  | 51 (68.9) | 4 (10.8) | 15 (40.5) | 18 (48.7) |  | Additive | 0.6510 | 1.24 (0.49-3.14) |
| Non-relapse |  |  |  | 18 (64.3) | 1 (7.1) | 8 (57.1) | 5 (35.7) |  | Dominant | 0.6966 | 0.63 (0.07-6.15) |
|  |  |  |  |  |  |  |  |  | Recessive | 0.4099 | 1.71 (0.48-6.03) |
| rs2523675 | 31468255 | 2.4 kb telomeric | rs2244546 | T | CC | CT | TT | 0.0268 | Allelic | 0.0328 | 2.75 (1.09-6.93) |
| Relapse |  | of HCP5 |  | 37 (50.0) | 13 (35.1) | 11 (29.7) | 13 (35.1) |  | Additive | 0.0600 | 2.30 (0.97-5.44) |
| Non-relapse |  |  |  | 8 (26.7) | 7 (46.7) | 8 (53.3) | . |  | Dominant | 0.4404 | 1.62 (0.48-5.43) |
|  |  |  |  |  |  |  |  |  | Recessive | 0.9554 | N/A |
| rs2518028 | 31468270 | 2.5 kb telomeric | rs2244546 | G | AA | AG | GG | 0.0233 | Allelic | 0.0684 | 2.91 (0.93-9.14) |
| Relapse |  | of HCP5 |  | 67 (90.5) | 1 (2.7) | 5 (13.5) | 31 (83.8) |  | Additive | 0.0748 | 3.00 (0.9-9.95) |
| Non-relapse |  |  |  | 23 (76.7) | . | 7 (46.7) | 8 (53.3) |  | Dominant | 0.9880 | N/A |
|  |  |  |  |  |  |  |  |  | Recessive | 0.0272 | 4.52 (1.19-17.13) |
| rs141431529 | 31468278 | 2.5 kb telomeric | rs2244546 | T | GG | GT | TT | 0.6743 | Allelic | 0.9736 | N/A |
| Relapse |  | of HCP5 |  | 5 (6.8) | 33 (89.2) | 3 (8.1) | 1 (2.7) |  | Additive | 0.9585 | N/A |
| Non-relapse |  |  |  | . | 14 (100.0) | . | . |  | Dominant | 0.9763 | N/A |
|  |  |  |  |  |  |  |  |  | Recessive | 0.9822 | N/A |
| ^1^ Assembly version: GRCh37.p13. ^2^ The sequenced SNPs were selected and studied based on the transplant determinants identified by Petersdorf *et al*. | | | | | | | | | | | |
