# Supplementary material for: Single nucleotide polymorphisms within HLA region are associated with disease relapse for patients with unrelated cord blood transplantation
Source: PeerJ. 2018 Aug 2;6:e5228. doi: 10.7717/peerj.5228 (PMC6076982; doi:10.7717/peerj.5228)
Supplement: Supplemental Information 9 — The association of group 2 SNPs with the risk of relapse for patients with unrelated CBT exact test. [file peerj-06-5228-s009.docx]

Supplementary Table S5. The association of group 2 SNPs with the risk of relapse for patients with unrelated CBT.

| **SNP** | **Physical position^1^ (bp)** | **Gene/location** | **Source^2^** | **Allele frequency**  **Number of allele (%)** | **Genotype frequency**  **Number of patients (%)** | | | **P** | **Model** | **P** | **OR** |
| --- | --- | --- | --- | --- | --- | --- | --- | --- | --- | --- | --- |
| rs543293268 | 31635041 | BAT2/PRRC2A, | rs11538264 | G | CC | CG | GG | N/A | Allelic | N/A | N/A |
| Relapse |  | intron |  | . | 38 (100.0) | . | . |  | Additive | N/A | N/A |
| Non-relapse |  |  |  | . | 15 (100.0) | . | . |  | Dominant | N/A | N/A |
|  |  |  |  |  |  |  |  |  | Recessive | N/A | N/A |
| rs17207239 | 31635117 | BAT2/PRRC2A, | rs11538264 | C | CC | CG | GG | 0.3088 | Allelic | 0.9761 | N/A |
| Relapse |  | intron |  | 4 (5.3) | . | 4 (10.5) | 34 (89.5) |  | Additive | 0.9760 | N/A |
| Non-relapse |  |  |  | . | . | . | 15 (100.0) |  | Dominant | 0.9760 | N/A |
|  |  |  |  |  |  |  |  |  | Recessive | N/A | N/A |
| rs1046089 | 31635190 | BAT2/PRRC2A, | rs11538264 | G | AA | AG | GG | 0.3480 | Allelic | 0.1218 | 2.00 (0.83-4.81) |
| Relapse |  | intron |  | 55 (72.4) | 3 (7.9) | 15 (39.5) | 20 (52.6) |  | Additive | 0.1355 | 1.97 (0.81-4.76) |
| Non-relapse |  |  |  | 17 (56.7) | 3 (20.0) | 7 (46.7) | 5 (33.3) |  | Dominant | 0.2251 | 2.92 (0.52-16.3) |
|  |  |  |  |  |  |  |  |  | Recessive | 0.2099 | 2.22 (0.64-7.69) |
| rs532278148 | 31635570 | BAT2/PRRC2A, | rs11538264 | T | CC | CT | TT | 0.2856 | Allelic | 0.9877 | N/A |
| Relapse |  | intron |  | 76 (100.0) | . | . | 38 (100.0) |  | Additive | 0.9811 | N/A |
| Non-relapse |  |  |  | 29 (96.7) | . | 1 (6.7) | 14 (93.3) |  | Dominant | N/A | N/A |
|  |  |  |  |  |  |  |  |  | Recessive | 0.9811 | N/A |
| rs115028652 | 31635676 | BAT2/PRRC2A, | rs11538264 | T | CC | CT | TT | 1.0000 | Allelic | 0.9881 | N/A |
| Relapse |  | intron |  | 1 (1.3) | 37 (97.4) | 1 (2.6) | . |  | Additive | 0.9881 | N/A |
| Non-relapse |  |  |  | . | 15 (100.0) | . | . |  | Dominant | 0.9881 | N/A |
|  |  |  |  |  |  |  |  |  | Recessive | N/A | N/A |
| rs2844463 | 31647390 | BAT3/BAG6, | rs10484558 | C | CC | CT | TT | 0.1637 | Allelic | 0.2240 | 1.88 (0.68-5.15) |
| Relapse |  | intron |  | 61 (82.4) | 25 (67.6) | 11 (29.7) | 1 (2.7) |  | Additive | 0.1941 | 2.13 (0.68-6.63) |
| Non-relapse |  |  |  | 20 (71.4) | 6 (42.9) | 8 (57.1) | . |  | Dominant | 0.9822 | N/A |
|  |  |  |  |  |  |  |  |  | Recessive | 0.1128 | 2.78 (0.79-9.76) |
| rs180712068 | 31647531 | BAT3/BAG6, | rs10484558 | G | CC | CG | GG | N/A | Allelic | N/A | N/A |
| Relapse |  | intron |  | . | 38 (100.0) | . | . |  | Additive | N/A | N/A |
| Non-relapse |  |  |  | . | 14 (100.0) | . | . |  | Dominant | N/A | N/A |
|  |  |  |  |  |  |  |  |  | Recessive | N/A | N/A |
| rs9276982 | 33010438 | 826 bp centromeric | rs429916 | G | AA | AG | GG | 0.0376 | Allelic | 0.0674 | 2.71 (0.94-7.83) |
| Relapse |  | of HLA-DOA |  | 67 (88.2) | 1 (2.6) | 7 (18.4) | 30 (79.0) |  | Additive | 0.0651 | 2.99 (0.94-9.5) |
| Non-relapse |  |  |  | 22 (73.3) | . | 8 (53.3) | 7 (46.7) |  | Dominant | 0.9881 | N/A |
|  |  |  |  |  |  |  |  |  | Recessive | 0.0258 | 4.29 (1.2-15.31) |
| rs71565361 | 33010551 | 939 bp centromeric | rs429916 | C | AA | AC | CC | 1.0000 | Allelic | 0.8620 | 1.24 (0.11-14.05) |
| Relapse |  | of HLA-DOA |  | 72 (97.3) | . | 2 (5.4) | 35 (94.6) |  | Additive | 0.8600 | 1.25 (0.11-14.73) |
| Non-relapse |  |  |  | 29 (96.7) | . | 1 (6.7) | 14 (93.3) |  | Dominant | N/A | N/A |
|  |  |  |  |  |  |  |  |  | Recessive | 0.8600 | 1.25 (0.11-14.73) |
| rs79327197 | 33010635 | 1 kb centromeric | rs429916 | A | AA | AG | GG | 0.0647 | Allelic | 0.0715 | 8.33 (0.84-82.59) |
| Relapse |  | Of HLA-DOA |  | 75 (98.7) | 37 (97.4) | 1 (2.6) | . |  | Additive | 0.0641 | 9.25 (0.89-96.3) |
| Non-relapse |  |  |  | 27 (90.0) | 12 (80.0) | 3 (20.0) | . |  | Dominant | N/A | N/A |
|  |  |  |  |  |  |  |  |  | Recessive | 0.0641 | 9.25 (0.89-96.3) |
| rs151190962 | 33010881 | 1.3 kb centromeric | rs429916 | - | AA | A- | -- | 1.0000 | Allelic | 0.9880 | N/A |
| Relapse |  | of HLA-DOA |  | 1 (1.4) | 36 (97.3) | 1 (2.7) | . |  | Additive | 0.9880 | N/A |
| Non-relapse |  |  |  | . | 15 (100.0) | . | . |  | Dominant | 0.9880 | N/A |
|  |  |  |  |  |  |  |  |  | Recessive | N/A | N/A |
| rs9282369^3^ | 33011011 | 1.4 kb centromeric | rs429916 | T | TT | T- | -- | 0.2445 | Allelic | 0.8591 | 1.08 (0.46-2.55) |
| Relapse | 33011012 | of HLA-DOA |  | 31 (41.9) | 12 (32.4) | 7 (18.9) | 18 (48.7) |  | Additive | 0.8924 | 1.05 (0.54-2.02) |
| Non-relapse |  |  |  | 12 (40.0) | 6 (40.0) | . | 9 (60.0) |  | Dominant | 0.4595 | 1.58 (0.47-5.32) |
|  |  |  |  |  |  |  |  |  | Recessive | 0.6040 | 0.72 (0.21-2.48) |
| rs2009658 | 31538244 | 1.6 kb telomeric | rs915654 | C | CC | CG | GG | 0.5289 | Allelic | 0.5004 | 1.43 (0.51-4.01) |
| Relapse |  | of LTA |  | 61 (82.4) | 25 (67.7) | 11 (29.7) | 1 (2.7) |  | Additive | 0.4750 | 1.50 (0.49-4.56) |
| Non-relapse |  |  |  | 23 (76.7) | 8 (53.3) | 7 (46.7) | . |  | Dominant | 0.9880 | N/A |
|  |  |  |  |  |  |  |  |  | Recessive | 0.3370 | 1.82 (0.54-6.17) |
| rs736160 | 31538496 | 1.3 kb telomeric | rs915654 | T | AA | AT | TT | 0.2856 | Allelic | 0.9877 | N/A |
| Relapse | 31538497 | of LTA |  | 76 (100.0) | . | . | 38 (100.0) |  | Additive | 0.9811 | N/A |
| Non-relapse |  |  |  | 29 (96.7) | . | 1 (6.7) | 14 (93.3) |  | Dominant | N/A | N/A |
|  |  |  |  |  |  |  |  |  | Recessive | 0.9811 | N/A |
| rs915654 | 31538497 | 1.3 kb telomeric | rs915654 | A | AA | AT | TT | 0.3523 | Allelic | 0.8112 | 1.11 (0.46-2.67) |
| Relapse |  | of LTA |  | 50 (65.8) | 15 (39.5) | 20 (52.6) | 3 (7.9) |  | Additive | 0.8076 | 1.12 (0.46-2.74) |
| Non-relapse |  |  |  | 19 (63.3) | 7 (46.7) | 5 (33.3) | 3 (20.0) |  | Dominant | 0.2251 | 2.92 (0.52-16.3) |
|  |  |  |  |  |  |  |  |  | Recessive | 0.6326 | 0.75 (0.22-2.47) |
| rs371621895 | 31809997 | HSPA1L, | rs2075800 | G | GG | G- | -- | 0.3048 | Allelic | 0.2012 | 2.13 (0.67-6.72) |
| Relapse |  | exon |  | 68 (89.5) | 30 (79.0) | 8 (21.1) | . |  | Additive | 0.2085 | 2.13 (0.66-6.89) |
| Non-relapse |  |  |  | 24 (80.0) | 10 (66.7) | 4 (26.6) | 1 (6.7) |  | Dominant | 0.9811 | N/A |
|  |  |  |  |  |  |  |  |  | Recessive | 0.3530 | 1.88 (0.5-7.02) |
| rs2075800 | 31810169 | HSPA1L, | rs2075800 | C | CC | CT | TT | 0.4704 | Allelic | 0.2592 | 1.68 (0.69-4.09) |
| Relapse |  | exon |  | 31 (45.6) | 6 (17.7) | 19 (55.9) | 9 (26.5) |  | Additive | 0.2294 | 1.83 (0.69-4.87) |
| Non-relapse |  |  |  | 10 (33.3) | 1 (6.7) | 8 (53.3) | 6 (40.0) |  | Dominant | 0.3467 | 1.85 (0.52-6.64) |
|  |  |  |  |  |  |  |  |  | Recessive | 0.3304 | 3.00 (0.33-27.09) |
| rs2227956 | 31810495 | HSPA1L, | rs2075800 | T | AA | AT | TT | 0.7467 | Allelic | 0.9022 | 1.07 (0.35-3.3) |
| Relapse |  | exon |  | 14 (18.9) | 24 (64.9) | 12 (32.4) | 1 (2.7) |  | Additive | 0.9036 | 1.07 (0.35-3.24) |
| Non-relapse |  |  |  | 5 (17.9) | 10 (71.4) | 3 (21.4) | 1 (7.1) |  | Dominant | 0.6579 | 1.35 (0.36-5.14) |
|  |  |  |  |  |  |  |  |  | Recessive | 0.4829 | 0.36 (0.02-6.12) |
| ^1^ Assembly version: GRCh37.p13. ^2^ The sequenced SNPs were selected and studied based on the transplant determinants identified by Petersdorf *et al*. Deviated from the Hardy-Weinberg equilibrium (HWE) with P < 5 x 10^-4^. | | | | | | | | | | | |
