# Supplementary material for: Single nucleotide polymorphisms within HLA region are associated with disease relapse for patients with unrelated cord blood transplantation
Source: PeerJ. 2018 Aug 2;6:e5228. doi: 10.7717/peerj.5228 (PMC6076982; doi:10.7717/peerj.5228)
Supplement: Supplemental Information 10 — The association of group 3 SNPs with the risk of relapse for patients with unrelated CBT as analyzed by Chi-square test and Fisher’s exact test. [file peerj-06-5228-s010.docx]

Supplemental Table S6. The association of group 3 SNPs with the risk of relapse for patients with unrelated CBT as analyzed by Chi-square test and Fisher’s exact test.

| **SNP** | **Physical position^1^ (bp)** | **Gene/location** | **Source^2^** | **Genotypes of donor-recipient Pairs**  **Number of patients (%)** | | **Chi-square Test P** | **Fisher's exact Test P** |
| --- | --- | --- | --- | --- | --- | --- | --- |
|  |  |  |  | Matched | Not Matched |  |  |
| rs3130048 | 31645962 | BAG6, | rs2242656 |  |  | 0.5300 | 0.7063 |
| Relapse |  | Intron |  | 29 (76.3) | 9 (23.7) |  |  |
| Non-relapse |  |  |  | 11 (84.6) | 2 (15.4) |  |  |
|  |  |  |  |  |  |  |  |
| rs2844464 | 31646214 | BAG6, | rs2242656 |  |  | 0.8640 | 1 |
| Relapse |  | Intron |  | 29 (76.3) | 9 (23.7) |  |  |
| Non-relapse |  |  |  | 11 (78.6) | 3 (21.4) |  |  |
|  |  |  |  |  |  |  |  |
| rs2242656 | 31646325 | BAG6, | rs2242656 |  |  | 0.8640 | 1 |
| Relapse |  | Intron |  | 29 (76.3) | 9 (23.7) |  |  |
| Non-relapse |  |  |  | 11 (78.6) | 3 (21.4) |  |  |
|  |  |  |  |  |  |  |  |
| rs3830076 | 32128467 | 240 bp telomeric | rs3830076 |  |  | 0.2053 | 0.2691 |
| Relapse |  | of FKBPL |  | 29 (82.9) | 6 (17.1) |  |  |
| Non-relapse |  |  |  | 10 (66.7) | 5 (33.3) |  |  |
|  |  |  |  |  |  |  |  |
| rs11244 | 32812947 | HLA-DOB, | rs2071479 |  |  | 0.5266 | 0.7284 |
| Relapse |  | 3’UTR |  | 25 (71.4) | 10 (28.6) |  |  |
| Non-relapse |  |  |  | 12 (80.0) | 3 (20.0) |  |  |
|  |  |  |  |  |  |  |  |
| rs2070120 | 32813137 | HLA-DOB, | rs2071479 |  |  | 0.9291 | 1 |
| Relapse |  | 3’UTR |  | 30 (85.7) | 5 (14.3) |  |  |
| Non-relapse |  |  |  | 13 (86.7) | 2 (13.3) |  |  |
|  |  |  |  |  |  |  |  |
| rs41258084 | 32813180 | HLA-DOB, | rs2071479 |  |  | 0.4234 | 0.4150 |
| Relapse |  | 3’UTR |  | 31 (88.6) | 4 (11.4) |  |  |
| Non-relapse |  |  |  | 12 (80.0) | 3 (20.0) |  |  |
|  |  |  |  |  |  |  |  |
| rs17220087 | 32813299 | HLA-DOB, | rs2071479 |  |  | 0.4474 | 0.6540 |
| Relapse |  | intron |  | 30 (85.7) | 5 (14.3) |  |  |
| Non-relapse |  |  |  | 14 (93.3) | 1 (6.7) |  |  |
|  |  |  |  |  |  |  |  |
| rs2071479 | 32813335 | HLA-DOB, | rs2071479 |  |  | 0.5287 | 0.5143 |
| Relapse |  | intron |  | 34 (97.1) | 1 (2.9) |  |  |
| Non-relapse |  |  |  | 14 (93.3) | 1 (6.7) |  |  |
|  |  |  |  |  |  |  |  |
| rs107822 | 33207798 | 711 bp telomeric | rs107822 |  |  | 0.7555 | 1 |
| Relapse |  | of RING1 |  | 16 (48.5) | 17 (51.5) |  |  |
| Non-relapse |  |  |  | 8 (53.3) | 7 (46.7) |  |  |
|  |  |  |  |  |  |  |  |
| rs213210 | 33208047 | 462 bp telomeric | rs107822 |  |  | 0.1698 | 0.2080 |
| Relapse |  | of RING1 |  | 10 (32.3) | 21 (67.7) |  |  |
| Non-relapse |  |  |  | 8 (53.3) | 7 (46.7) |  |  |
|  |  |  |  |  |  |  |  |
| rs435766 | 29939852 | MICD | rs2523957 |  |  | 0.4736 | 0.5335 |
| Relapse |  |  |  | 22 (62.9) | 13 (37.1) |  |  |
| Non-relapse |  |  |  | 11 (73.3) | 4 (26.7) |  |  |
|  |  |  |  |  |  |  |  |
| rs380924 | 29939885 | MICD | rs2523957 |  |  | 0.4736 | 0.5335 |
| Relapse |  |  |  | 22 (62.9) | 13 (37.1) |  |  |
| Non-relapse |  |  |  | 11 (73.3) | 4 (26.7) |  |  |
|  |  |  |  |  |  |  |  |
| rs1264813 | 29939900 | MICD | rs2523957 |  |  | 0.1615 | 0.2936 |
| Relapse |  |  |  | 23 (65.7) | 12 (34.3) |  |  |
| Non-relapse |  |  |  | 12 (85.7) | 2 (14.3) |  |  |
|  |  |  |  |  |  |  |  |
| rs2523960 | 29939952 | MICD | rs2523957 |  |  | 0.6646 | 1 |
| Relapse |  |  |  | 26 (74.3) | 9 (25.7) |  |  |
| Non-relapse |  |  |  | 12 (80.0) | 3 (20.0) |  |  |
|  |  |  |  |  |  |  |  |
| rs2523959 | 29939978 | MICD | rs2523957 |  |  | 0.6646 | 1 |
| Relapse |  |  |  | 26 (74.3) | 9 (25.7) |  |  |
| Non-relapse |  |  |  | 12 (80.0) | 3 (20.0) |  |  |
|  |  |  |  |  |  |  |  |
| rs2523958 | 29940202 | MICD | rs2523957 |  |  | 0.7363 | 1 |
| Relapse |  |  |  | 24 (68.6) | 11 (31.4) |  |  |
| Non-relapse |  |  |  | 11 (73.3) | 4 (26.7) |  |  |
|  |  |  |  |  |  |  |  |
| rs2523957 | 29940260 | MICD | rs2523957 |  |  | 0.8388 | 1 |
| Relapse |  |  |  | 21 (63.6) | 12 (36.4) |  |  |
| Non-relapse |  |  |  | 10 (66.7) | 5 (33.3) |  |  |
|  |  |  |  |  |  |  |  |
| rs5009448 | 29940488 | MICD | rs2523957 |  |  | 0.2959 | 0.3512 |
| Relapse |  |  |  | 19 (57.6) | 14 (42.4) |  |  |
| Non-relapse |  |  |  | 11 (73.3) | 4 (26.7) |  |  |
|  |  |  |  |  |  |  |  |
| rs209132 | 28899705 | 3.3 kb telomeric | rs209130 |  |  | 0.4626 | 0.5431 |
| Relapse |  | of TRIM27 |  | 20 (55.6) | 16 (44.4) |  |  |
| Non-relapse |  |  |  | 10 (66.7) | 5 (33.3) |  |  |
|  |  |  |  |  |  |  |  |
| rs209131 | 28899978 | 3 kb telomeric | rs209130 |  |  | 0.5145 | 0.5542 |
| Relapse |  | of TRIM27 |  | 18 (50.0) | 18 (50.0) |  |  |
| Non-relapse |  |  |  | 6 (40.0) | 9 (60.0) |  |  |
| ^1^ Assembly version: GRCh37.p13. ^2^ The sequenced SNPs were selected and studied based on the transplant determinants identified by Petersdorf *et al.* | | | | | | | |
