# Supplementary material for: Single nucleotide polymorphisms within HLA region are associated with disease relapse for patients with unrelated cord blood transplantation
Source: PeerJ. 2018 Aug 2;6:e5228. doi: 10.7717/peerj.5228 (PMC6076982; doi:10.7717/peerj.5228)
Supplement: Supplemental Information 11 — The association of group 3 SNPs with the risk of relapse for patients with unrelated CBT as analyzed by genotypic test. [file peerj-06-5228-s011.docx]

Supplementary Table S7. The association of group 3 SNPs with the risk of relapse for patients with unrelated CBT as analyzed by genotypic test.

| **SNP** | **Physical position^1^ (bp)** | **Gene/location** | **Source^2^** | **Donor's genotype frequency**  **Number of patients (%)** | | | **P** | **Recipient’s genotype frequency**  **Number of patients (%)** | | | **P** |
| --- | --- | --- | --- | --- | --- | --- | --- | --- | --- | --- | --- |
|  |  |  |  |  |  |  |  |  |  |  |  |
| rs3130048 | 31645962 | BAG6, | rs2242656 | CC | CT | TT | 1.0000 | CC | CT | TT | 0.8105 |
| Relapse |  | intron |  | 4 (10.5) | 18 (47.3) | 16 (42.1) |  | 1 (2.6) | 20 (52.6) | 17 (44.7) |  |
| Non-relapse |  |  |  | 1 (7.7) | 6 (46.1) | 6 (46.2) |  | 1 (6.7) | 9 (60.0) | 5 (33.3) |  |
|  |  |  |  |  |  |  |  |  |  |  |  |
| rs2844464 | 31646214 | BAG6, | rs2242656 | CC | CT |  | 0.1128 | CC | CT |  | 0.1887 |
| Relapse |  | intron |  | 33 (86.8) | 5 (13.2) |  |  | 30 (79.0) | 8 (21.1) |  |  |
| Non-relapse |  |  |  | 9 (64.3) | 5 (35.7) |  |  | 9 (60.0) | 6 (40.0) |  |  |
|  |  |  |  |  |  |  |  |  |  |  |  |
| rs2242656 | 31646325 | BAG6, | rs2242656 | AA | AG |  | 0.1122 | AA | AG |  | 0.1831 |
| Relapse |  | intron |  | 33 (86.8) | 5 (13.2) |  |  | 30 (79.0) | 8 (21.1) |  |  |
| Non-relapse |  |  |  | 9 (64.3) | 5 (35.7) |  |  | 9 (60.0) | 6 (40.0) |  |  |
|  |  |  |  |  |  |  |  |  |  |  |  |
| rs3830076 | 32128467 | 240 bp telomeric | rs3830076 | AG | GG |  | 0.6609 | AG | GG |  | 0.2154 |
| Relapse |  | of FKBPL |  | 4 (11.4) | 31 (88.6) |  |  | 4 (10.8) | 33 (89.2) |  |  |
| Non-relapse |  |  |  | 3 (20.0) | 12 (80.0) |  |  | 4 (26.7) | 11 (73.3) |  |  |
|  |  |  |  |  |  |  |  |  |  |  |  |
| rs11244 | 32812947 | HLA-DOB, | rs2071479 | CC | CT | TT | 0.8881 | CC | CT | TT | 0.8372 |
| Relapse |  | 3’ UTR |  | 19 (54.3) | 14 (40.0) | 2 (5.7) |  | 21 (56.8) | 15 (40.5) | 1 (2.7) |  |
| Non-relapse |  |  |  | 7 (46.7) | 7 (46.7) | 1 (6.7) |  | 8 (53.3) | 7 (46.7) | 0 (0.0) |  |
|  |  |  |  |  |  |  |  |  |  |  |  |
| rs2070120 | 32813137 | HLA-DOB, | rs2071479 | CC | CT |  | 1.0000 | CC | CT |  | 1.0000 |
| Relapse |  | 3’ UTR |  | 34 (97.1) | 1 (2.9) |  |  | 31 (83.8) | 6 (16.2) |  |  |
| Non-relapse |  |  |  | 15 (100.0) | 0 (0.0) |  |  | 13 (86.7) | 2 (13.3) |  |  |
|  |  |  |  |  |  |  |  |  |  |  |  |
| rs41258084 | 32813180 | HLA-DOB, | rs2071479 | CC | CT |  | 0.3453 | CC | CT | TT | 0.0615 |
| Relapse |  | 3’ UTR |  | 32 (91.4) | 3 (8.6) |  |  | 36 (97.3) | 1 (2.7) | 0 (0.0) |  |
| Non-relapse |  |  |  | 12 (80.0) | 3 (20.0) |  |  | 12 (80.0) | 2(13.3) | 1 (6.7) |  |
|  |  |  |  |  |  |  |  |  |  |  |  |
| rs17220087 | 32813299 | HLA-DOB, | rs2071479 | AC | CC |  | 1.0000 | AC | CC |  | 0.4378 |
| Relapse |  | intron |  | 1 (2.9) | 34 (97.1) |  |  | 6 (16.2) | 31 (83.8) |  |  |
| Non-relapse |  |  |  | 0 (0.0) | 15 (100.0) |  |  | 1 (6.7) | 14 (93.3) |  |  |
|  |  |  |  |  |  |  |  |  |  |  |  |
| rs2071479 | 32813335 | HLA-DOB, | rs2071479 | CC | CT |  | 0.0077 | CC | CT |  | 0.0685 |
| Relapse |  | intron |  | 35 (100.0) | 0 (0.0) |  |  | 36 (97.3) | 1 (2.7) |  |  |
| Non-relapse |  |  |  | 11 (73.3) | 4 (26.7) |  |  | 12 (80.0) | 3 (20.0) |  |  |
|  |  |  |  |  |  |  |  |  |  |  |  |
| rs107822 | 33207798 | 711 bp telomeric | rs107822 | AA | AG | GG | 0.8303 | AA | AG | GG | 0.0583 |
| Relapse |  | of RING1 |  | 15 (42.9) | 16 (45.7) | 4 (11.4) |  | 25 (69.4) | 9 (25.0) | 2 (5.6) |  |
| Non-relapse |  |  |  | 6 (40.0) | 8 (53.3) | 1 (6.7) |  | 5 (33.3) | 9 (60.0) | 1 (6.7) |  |
|  |  |  |  |  |  |  |  |  |  |  |  |
| rs213210 | 33208047 | 462 bp telomeric | rs107822 | CC | CT | TT | 0.9247 | CC | CT | TT | 0.0992 |
| Relapse |  | of RING1 |  | 9 (26.5) | 16 (47.1) | 9 (26.5) |  | 18 (51.4) | 14 (40.0) | 3 (8.6) |  |
| Non-relapse |  |  |  | 4 (26.7) | 8 (53.3) | 3 (20.0) |  | 3 (20.0) | 10 (66.7) | 2 (13.3) |  |
|  |  |  |  |  |  |  |  |  |  |  |  |
| rs435766 | 29972075 | MICD | rs2523957 | CC | CT | TT | 0.3560 | CC | CT | TT | 0.0130 |
| Relapse |  |  |  | 11 (30.6) | 17 (47.2) | 8 (22.2) |  | 14 (37.8) | 11 (29.7) | 12 (32.4) |  |
| Non-relapse |  |  |  | 4 (26.7) | 10 (66.7) | 1 (6.7) |  | 5 (33.3) | 10 (66.7) | 0 (0.0) |  |
|  |  |  |  |  |  |  |  |  |  |  |  |
| rs380924 | 29972108 | MICD | rs2523957 | CC | CT | TT | 0.3548 | CC | CT | TT | 0.0320 |
| Relapse |  |  |  | 8 (22.2) | 17 (47.2) | 11 (30.6) |  | 12 (32.4) | 11 (29.7) | 14 (37.8) |  |
| Non-relapse |  |  |  | 1 (6.7) | 10 (66.7) | 4 (26.7) |  | 1 (6.7) | 10 (66.7) | 4 (26.7) |  |
|  |  |  |  |  |  |  |  |  |  |  |  |
| rs1264813 | 29972123 | MICD | rs2523957 | AA | AG | GG | 0.5210 | AA | AG | GG | 0.8892 |
| Relapse |  |  |  | 3 (8.3) | 8 (22.2) | 25 (69.4) |  | 1 (2.7) | 10 (27.0) | 26 (70.3) |  |
| Non-relapse |  |  |  | 0 (0.0) | 5 (33.3) | 10 (66.7) |  | 1 (6.7) | 5 (33.3) | 9 (60.0) |  |
|  |  |  |  |  |  |  |  |  |  |  |  |
| rs2523960 | 29972175 | MICD | rs2523957 | AA | AG | GG | 0.2328 | AA | AG | GG | 0.8898 |
| Relapse |  |  |  | 26 (72.2) | 7 (19.4) | 3 (8.3) |  | 26 (70.3) | 10 (27.0) | 1 (2.7) |  |
| Non-relapse |  |  |  | 9 (60.0) | 6 (40.0) | 0 (0.0) |  | 9 (60.0) | 5 (33.3) | 1 (6.7) |  |
|  |  |  |  |  |  |  |  |  |  |  |  |
| rs2523959 | 29972201 | MICD | rs2523957 | GG | GT | TT | 0.1384 | GG | GT | TT | 0.8940 |
| Relapse |  |  |  | 26 (72.2) | 6 (16.7) | 4 (11.1) |  | 25 (67.6) | 11 (29.7) | 1 (2.7) |  |
| Non-relapse |  |  |  | 9 (60.0) | 6 (40.0) | 0 (0.0) |  | 9 (60.0) | 5 (33.3) | 1 (6.7) |  |
|  |  |  |  |  |  |  |  |  |  |  |  |
| rs2523958 | 29972425 | MICD | rs2523957 | CC | CT | TT | 0.0445 | CC | CT | TT | 0.8819 |
| Relapse |  |  |  | 26 (72.2) | 6 (16.7) | 4 (11.1) |  | 26 (70.3) | 10 (27.0) | 1 (2.7) |  |
| Non-relapse |  |  |  | 8 (53.3) | 7 (46.7) | 0 (0.0) |  | 9 (60.0) | 5 (33.3) | 1 (6.7) |  |
|  |  |  |  |  |  |  |  |  |  |  |  |
| rs2523957 | 29972483 | MICD | rs2523957 | CC | CT | TT | 0.3634 | CC | CT | TT | 0.0813 |
| Relapse |  |  |  | 8 (22.2) | 17 (47.2) | 11 (30.6) |  | 12 (34.3) | 11 (31.4) | 12 (34.3) |  |
| Non-relapse |  |  |  | 1 (6.7) | 10 (66.7) | 4 (26.7) |  | 2 (13.3) | 10 (66.7) | 3 (20.0) |  |
|  |  |  |  |  |  |  |  |  |  |  |  |
| rs5009448 | 29972711 | MICD | rs2523957 | CC | CT | TT | 0.3245 | CC | CT | TT | 0.1727 |
| Relapse |  |  |  | 11 (30.6) | 19 (52.8) | 6 (16.7) |  | 15 (42.9) | 13 (37.1) | 7 (20.0) |  |
| Non-relapse |  |  |  | 8 (53.3) | 6 (40.0) | 1 (6.7) |  | 7 (46.7) | 8 (53.3) | 0 (0.0) |  |
|  |  |  |  |  |  |  |  |  |  |  |  |
| rs209132 | 28899705 | 3.3 kb telomeric | rs209130 | CC | CT | TT | 0.8339 | CC | CT | TT | 0.5208 |
| Relapse |  | of TRIM27 |  | 20 (52.6) | 13 (34.2) | 5 (13.2) |  | 22 (61.1) | 12 (33.3) | 2 (5.6) |  |
| Non-relapse |  |  |  | 8 (53.3) | 6 (40.0) | 1 (6.7) |  | 7 (46.7) | 6 (40.0) | 2 (13.3) |  |
|  |  |  |  |  |  |  |  |  |  |  |  |
| rs209131 | 28899978 | 3 kb telomeric | rs209130 | CC | CT | TT | 1.000 | CC | CT | TT | 0.0910 |
| Relapse |  | of TRIM27 |  | 8 (21.1) | 21 (55.3) | 9 (23.7) |  | 9 (25.0) | 21 (58.3) | 6 (16.7) |  |
| Non-relapse |  |  |  | 4 (26.7) | 8 (53.3) | 3 (20.0) |  | 3 (20.0) | 5 (33.3) | 7 (46.7) |  |
